# On the Impact of Mobility on Battery-Less RF Energy Harvesting System Performance

**DOI:** 10.3390/s18113597

**Published:** 2018-10-23

**Authors:** Bilal Munir, Vladimir Dyo

**Affiliations:** Department of Computer Science and Technology, University of Bedfordshire, Luton LU1 3JU, UK; bilal.munir@study.beds.ac.uk

**Keywords:** wireless sensor networks, energy harvesting, mobile computing, supercapacitors

## Abstract

The future of Internet of Things (IoT) envisions billions of sensors integrated with the physical environment. At the same time, recharging and replacing batteries on this infrastructure could result not only in high maintenance costs, but also large amounts of toxic waste due to the need to dispose of old batteries. Recently, battery-free sensor platforms have been developed that use supercapacitors as energy storage, promising maintenance-free and perpetual sensor operation. While prior work focused on supercapacitor characterization, modelling and supercapacitor-aware scheduling, the impact of mobility on capacitor charging and overall sensor application performance has been largely ignored. We show that supercapacitor size is critical for mobile system performance and that selecting an optimal value is not trivial: small capacitors charge quickly and enable the node to operate in low energy environments, but cannot support intensive tasks such as communication or reprogramming; increasing the capacitor size, on the other hand, enables the support for energy-intensive tasks, but may prevent the node from booting at all if the node navigates in a low energy area. The paper investigates this problem and proposes a hybrid storage solution that uses an adaptive learning algorithm to predict the amount of available ambient energy and dynamically switch between two capacitors depending on the environment. The evaluation based on extensive simulations and prototype measurements showed up to 40% and 80% improvement compared to a fixed-capacitor approach in terms of the amount of harvested energy and sensor coverage.

## 1. Introduction

Battery-free sensors gradually realize the vision of perpetual and maintenance-free sensing applications [1]. The energy harvesting sensors extract energy from the environment and store it in supercapacitors, which can operate forever without maintenance due to unlimited charge-discharge cycles [2]. As the amount of harvested energy depends on the environment, the sensor node can no longer be expected to operate continuously, but instead, will operate intermittently whenever energy becomes available, for example when the node moves into a high energy area.

Small supercapacitors are attractive for mobile systems because they charge and boot the battery-less sensor node in a matter of seconds whenever energy becomes available. This choice, however, prevents the sensor node from performing energy-intensive operations, such as communicating with a remote base station. Large capacitors, on the other hand, may take such a long time to charge, that the node may leave a high energy area long before the capacitor gets sufficient charge to start-up the node, let alone perform any useful task. Furthermore, as the node leaves the high energy area, the partial charge in the large supercapacitor dissipates rapidly without being used. Surprisingly, the design trade-offs in selecting an optimal storage capacity and the impact of mobility on the charging performance of intermittently-powered battery-less systems have not been described in the literature.

The supercapacitors differ from traditional batteries by low energy density and high self-discharge current: Once fully charged, the supercapacitors discharge at a much higher rate than a traditional battery. Prior work focused on supercapacitor characterization [3], modelling and supercapacitor-aware task scheduling [4]. The benefits of small capacitors for mobile applications and the capacitor-based energy harvesting sensor platform were proposed by [5]. Recently, Hester et al. [1] developed a federated energy storage concept, where each peripheral, such as radio, sensor or a microcontroller, is assigned a dedicated capacitor for increased reliability so that faulty or misconfigured peripherals do not affect the shared energy storage and thus the operation of the entire system. The paper does not, however, address the impact of mobility on charging and the application performance of battery-less sensors.

In this paper, we propose a novel dual capacitor energy storage platform that dynamically selects an appropriate energy storage based on the amount of ambient energy. The dynamic switching is not trivial since it requires tracking and predicting the amount of available energy in the environment. We show that the hybrid approach allows meeting the otherwise contradictory requirements by avoiding the design trade-offs present in traditional single-capacitor designs. We define a new performance metric, which we call usable harvested energy, as the amount of energy directly available to applications and show that it can be a fraction of total energy harvested by the node due to supercapacitor self-discharge current and residual charge. Finally, we evaluate the proposed platform through extensive simulations and a hardware prototype based on the PowerCast RF energy harvesting development kit [6]. To the best of our knowledge, this is the first work to investigate the impact of mobility on battery-less wireless sensors and to propose a working solution based on a novel energy prediction algorithm.

The rest of the paper is structured as follows. Section 2 and Section 3 contain a system model and describe the proposed approach including a hardware platform and a learning algorithm. Section 4 and Section 5 contain the results of simulation and prototype measurements, respectively. Section 6 reviews related work on supercapacitor-based energy harvesting sensor systems, and finally, Section 7 concludes the paper.

## 2. Energy Trade-Offs for Mobile Systems

Our system consists of mobile Radio Frequency (RF) energy harvesting sensor nodes deployed over a large geographical area and powered by dedicated RF power transmitters deployed at strategic locations. As the maximum transmit power of RF transmitters is limited by regulations, the charging range is small relative to distance between RF power transmitters, and the resulting charging network is assumed to be sparse. Whenever a sensor node approaches an RF power transmitter, it starts charging and boots if a sufficient amount of energy is harvested. As the node leaves an area with an RF power transmitter, it keeps operating until it uses up the available energy stored in the capacitor. Each RF charging station modulates a charging signal to embed additional information, such as station ID, which enables the mobile nodes to distinguish between different stations and therefore locations.

The amount of harvested energy depends on the distance to the charging station, transmit power and the amount of time the sensor spends near the charging station. The available wireless RF power on a receiving antenna of a sensor node is given by the Friis equation:

(1) $$P_{r}=\eta P_{t}G_{t}G_{r}{\left(\frac{\lambda }{4\pi R}\right)}^{2}$$

where $P_{t}$ is the transmit power, $G_{t}$ and $G_{r}$ are antenna gains for the transmitter and receiving antenna, respectively, λ is the wavelength, *R* is the distance between a charging station and a sensor node and η is the charging efficiency, which depends on the frequency band and the RF power level. The amount of harvested energy depends on the time the sensor node spends near the RF charging station; our experiments show that a 50-mF capacitor located at a distance of 3 m from a 3-W transmitter charges within 29 s.

**Figure 1:**
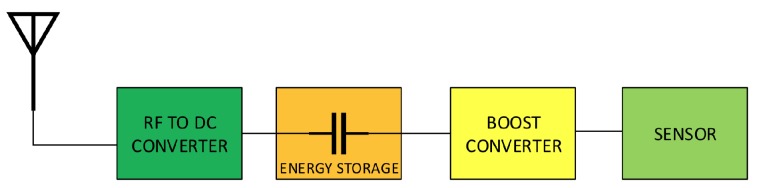
Block diagram of RF energy harvesting sensor node.

Figure 1 shows a block diagram of a battery-less RF energy harvesting system, which consists of an RF to DC converter, a boost converter and a supercapacitor. As the capacitor voltage varies with the amount of stored energy, a boost converter is used to supply a stable voltage necessary to the sensor node. The amount of energy stored by a supercapacitor is given as:

(2) $$E=CV^{2}/2$$

**Figure 2:**
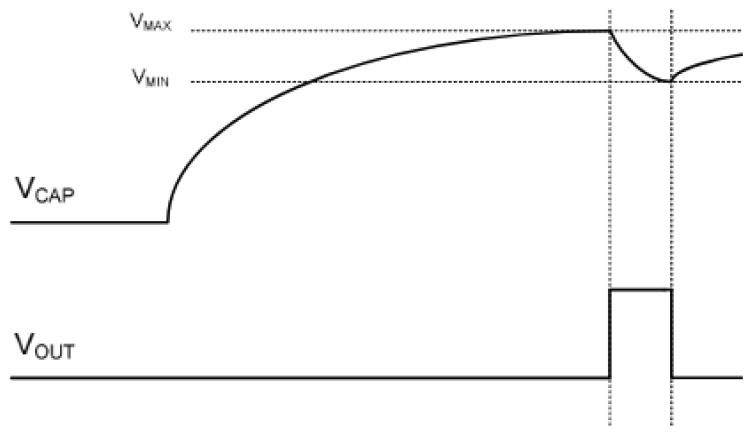
The timing diagram of capacitor charging. The sensor activates when the capacitor voltage reaches $V_{max}$ and keeps operating until the voltage drops to $V_{min}$ [6].

It is important to note that not all of the stored energy is actually usable because the boost converter requires a minimum voltage, $V_{min}$, to start up. In fact, modern energy harvesting systems are designed such that the voltage booster activates when the capacitor voltage reaches $V_{max}$ and keeps operating until the voltage drops to $V_{min}$, as shown in Figure 2. The hysteresis behaviour ensures that there is sufficient energy to reliably boot the node [7], i.e., $C\left(V_{max}^{2}-V_{min}^{2}\right)/2>E_{boot}$. At the same time, it leads to loss of energy in mobile scenarios as explained in the section below.

### 2.1. Charging Problem

As the amount of harvested energy depends on the environment, there is no guarantee that the capacitor voltage will be charged to $V_{max}$ within one charging session. Due to mobility, a sensor node may leave the charging station before a $V_{max}$ is reached, so any partial charge will gradually get dissipated before the node finds another energy source due to relatively the high self-discharge currents of capacitors, leading to energy wastage and inefficient operation. At $V_{max}$ = 1.25 and $V_{min}$ = 1.02, which is the standard operating range for PowerCast RF, the partial energy loss $E_{loss}=CV_{residual}^{2}/2$ represents 67%–100% of energy harvested by the capacitor. The exact figure depends on the node mobility pattern with the worst case scenario in which all harvested energy is lost between the individual charging sessions. While the loss is also linearly proportional to the capacitor value with larger capacitors leading to more loss (2), the relationship is more complex, as the node may never boot when operating on a large capacitor.

### 2.2. Keeping the Charge

As the node boots, the most energy-efficient way to utilise the energy is to consume it straight away to avoid energy loss due to capacitor leak current $E_{leak}$ and the minimum energy needed to operate the sensor node in the sleep mode $E_{sleep}$. The supercapacitor energy level at time *t* is modelled as:

(3) $$E\left(t+1\right)=E\left(t\right)+E_{harvested}\left(t\right)-E_{leak}\left(t\right)-E_{load}\left(t\right)$$

where $E_{load}$ denotes energy consumed by the sensor node and depends on the sensor operating mode, e.g., in case a sensor node is sleeping $E_{load}=E_{sleep}$. However, using energy immediately is not necessarily useful, as many sensor tasks require spreading the energy in time to maximise the utility, e.g., sensor coverage or operating time. Reducing the duty cycle in an attempt to spread the energy will lead to higher energy loss, $E_{leak}$, and consequently lower energy utilisation. Thus, the tasks that require long operating time or sensor coverage would benefit from a larger capacitor, which conflicts with the fast boot time requirement.

### 2.3. Task Size

Last but not least, the task size is an important factor in the design of the energy harvesting system. Even the most basic applications require the ability to combine light tasks, such as sensing or computation, with large tasks, such as communicating with other nodes or the base station. These tasks are often separated in time with light tasks dominating most of the time and larger tasks executed occasionally to upload sensed data or reprogram the node firmware. The light tasks may benefit from a small storage to quickly start up the node and execute a task. On the other hand, energy-intensive tasks require a larger storage, which again comes into conflict with the ability to boot quickly and charging efficiency.

To summarise, the design of a battery-less energy harvesting systems is driven by a number of trade-offs and sometimes contradicting requirements. The energy storage size is the key design parameter, which has implications on the charging problem, keeping the charge and maximum task size. In this paper, we argue that these challenges can be met by a hybrid storage solution, which combines several capacitors of various sizes.

## 3. Approach

The proposed RF energy harvesting sensor platform contains a hybrid storage containing two supercapacitors of various sizes. In mobile applications, where a node may have limited time passing through the RF charging station, it is critical that the sensor is able to charge its capacitor fully before it leaves the area. The small capacitor is used to boot quickly and perform at least a minimal task, such as sensing or sending a beacon. The large capacitor accumulates energy in high energy areas for energy-intensive tasks, such as communication with the base station. The proposed platform also incorporates a switching hardware, an energy prediction based on the Kalman filter and a switching algorithm, as described in the following subsections.

### 3.1. Hardware Platform

**Figure 3:**
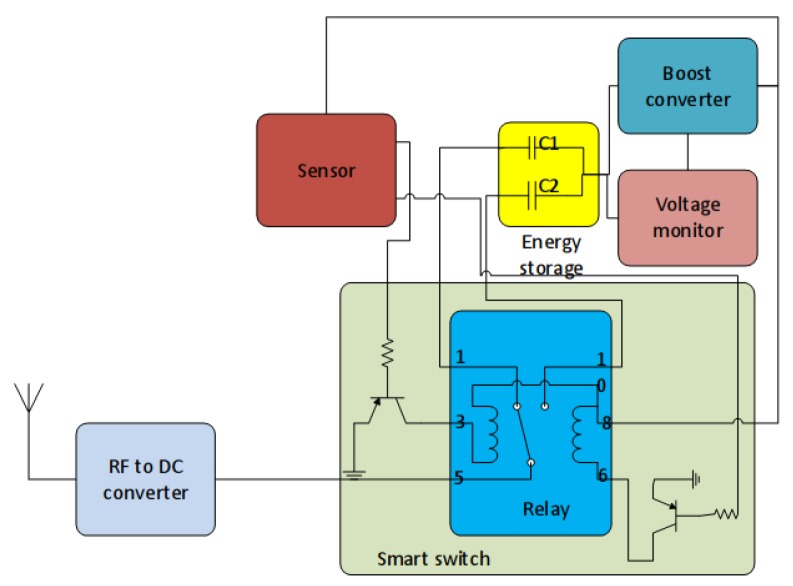
Smart switching circuit for supercapacitors.

The proposed platform consists of an energy harvesting sensor node, two capacitors and a switching circuit. The switching circuit consists of one bi-stable relay, two bipolar junction transistors (BJT) and two resistors, as shown in Figure 3. The P1 relay is bi-stable single pole double throw relay, which operates at 3 V and consumes 69 mW. The set and reset take 2 ms, which means it consumes 138 μJ energy per switch. The advantage of using a relay is that once set, it does not consume energy and maintains its state across the reboots. This is particularly suitable for sparse charging networks where the mobile node roams between high and low energy areas as the circuit does not consume energy once it moves into a certain area. The circuit is controlled by sensor node’s General Purpose Input Output (GPIO) ports using a switching algorithm as described in the following subsection.

### 3.2. Switching Algorithm

The key to efficient dual capacitor operation is an intelligent switching algorithm that dynamically switches between small and large capacitors depending on the situation. The amount of energy harvested by the node near an RF charging station largely depends on collocation time τ, i.e., the time the node spends near the station:

(4) $$E_{rx}=\int_{0}^{\tau }P_{r}dt$$

where $P_{r}$ is an instantaneous RF received power level.

In human- and animal-centric applications, the collocation time and the node mobility in general are well known to exhibit strong statistical patterns [8,9], which have been widely studied and exploited in the context of opportunistic routing protocols [10,11], mobility pattern [12] and social network analysis [9,13]. Delay-tolerant routing protocols exploit network statistical patterns to identify the next hop towards the destination, which is more likely to deliver the message. The work in social network analysis uses patterns in collocation time to gauge the strength of the social links and analyse users’ geographical preferences. This property of human- and animal-centric networks is also exploited in this work.

The main idea of the proposed switching algorithm is to learn the amount of harvestable energy in each location based on the average time the node spends near a charging station. This is based on the observation that human mobility is not random and exhibits strong statistical patterns. Consider for example a wearable application, where a mobile node charges from RF charging stations located throughout a university campus. When a person enters his or her office, there is a high probability that he or she will stay in that location for a longer period of time, $\tau_{1}$. However, when a user is passing by a building entrance, it is more likely to be just a short encounter, i.e., $\tau_{1}>\tau_{2}$.

The proposed algorithm works as follows. When a sensor node approaches a charging station, it starts sampling an RF signal strength at fixed time intervals $T_{s}$ and counts the total duration of time when the signal is above a threshold $P_{RF}>P_{thresh}$ to estimate the node collocation time $x_{A}\left(t\right)$ near the RF charging station *A*. The $P_{RF}$ is assumed to be constant during a timeslot $T_{s}$, which is reasonable for sufficiently small values of $T_{s}$. The node then predicts the amount of available energy as $E_{A}=P_{thresh}\times x_{A}\left(t\right)$. Since the energy is tracked only when the node is active and when the signal strength is above threshold $P_{thresh}$, the predicted $E_{A}$ will slightly underestimate the amount of available energy. This is not critical, as the priority is not to overestimate the available energy, which would cause the node to switch to a larger capacitor and remain inoperable until it moves to an area with high energy. The advantage of our collocation tracking technique is that it is simple and can be implemented on real hardware.

Since collocation time is random and may change every time a user visits a certain location, we use the Kalman filter to predict the future collocation time. The Kalman filter is computationally lightweight and requires minimum processing power and storage [14], which makes it very suitable for resource-constrained platforms. One of the main advantages of using the Kalman filter is that it does not require storing the entire history of past collocation times and instead uses a set of recursive equations to make a prediction. The collocation time near a charging station is modelled as a linear process.

(5) $$\begin{array}{c} x(t)=ax(t-1)+w(t) \end{array}$$

(6) $$\begin{array}{c} z(t)=hx(t)+v(t) \end{array}$$

where x(t) is a state estimate at time *t*, z(t) is the current measurement of the collocation time at time *t*, *a* is a state transition model, *h* is a measurement matrix and w(t) and v(t) are a process and measurement noise, respectively. For clarity, we dropped the subindex identifying the station ID from x(t) with all future references of x(t) referring to the collocation time of a particular charging station. As mentioned in the previous section, the node recognises a location by demodulating and extracting a station ID embedded into a charging signal, a functionality supported by energy harvesting development kits such as PowerCast [6]. The goal of the Kalman filter is to estimate a state x(t) from noisy observations z(t).

The Kalman filter operates recursively in two steps. The first step predicts the future value of collocation time $\hat{x}{\left(t\right)}^{-}$ based on past collocation history:

(7) $$\begin{array}{c} \hat{x}{\left(t\right)}^{-}=a\hat{x}\left(t-1\right)+w\left(t\right) \end{array}$$

(8) $$\begin{array}{c} p{\left(t\right)}^{-}=a^{2}p\left(t-1\right)+Q \end{array}$$

After observing the actual measurement z(t), the filter corrects the prediction and updates the model in an update step:

(9) $$\begin{array}{c} k\left(t\right)=\frac{hp{\left(t\right)}^{-}}{h^{2}p{\left(t\right)}^{-}+R} \end{array}$$

(10) $$\begin{array}{c} \hat{x}\left(t\right)=a\hat{x}{\left(t\right)}^{-}+k\left(t\right)\left(z\left(t\right)-h\hat{x}{\left(t\right)}^{-}\right) \end{array}$$

(11) $$\begin{array}{c} p\left(t\right)=p{\left(t\right)}^{-}\left(1-hk\left(t\right)\right) \end{array}$$

where p(t), *Q* and *R* are a posteriori error covariance, process and measurement noise covariance matrices, respectively; k(t) is a Kalman gain, which is the relative weight given to recent measurement and the current state estimate. For the scalar Kalman filter, *Q* and *R* correspond to a variance of the process and the variance of the measurement noise, respectively. We refer the reader to [14,15] for a detailed description of Kalman filter operation. The detailed steps of the algorithm are shown in Algorithm 1. Initially, the node boots on a small capacitor which enables it to operate in all environments and switches to a larger capacitor only if sufficient energy is available. The predicted amount of available energy is based on the expected collocation time x(t), and if it is sufficient to charge a larger capacitor, i.e., if $P_{thresh}\times x_{i}\left(t\right)\geq CV^{2}/2$, it initiates a switch. The sensor node keeps operating on a large capacitor after it moves away from the charging station until the voltage drops below threshold $V_{low}$, when it switches back to a small capacitor. As the capacitor voltage drops below $V_{min}$, the sensor node shuts down, and the residual energy in the capacitor is gradually dissipated. 

**Algorithm 1:** Switching algorithm.

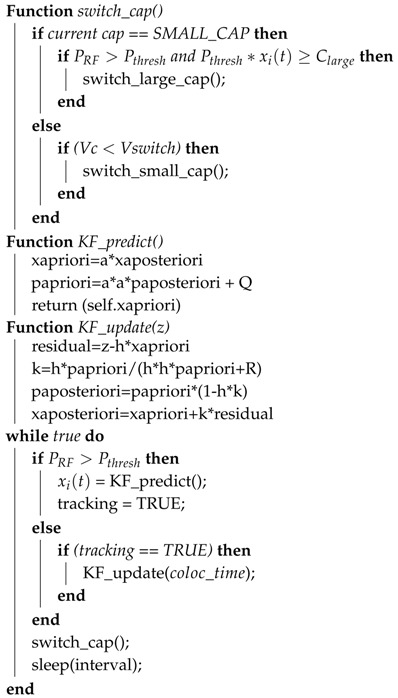



## 4. Simulations

The goal of the simulation is to evaluate the performance of the proposed approach for a typical sensing application, where a mobile node roams within a large area from one charging station to another and opportunistically senses data. To mimic practical scenarios, where RF charging stations are placed along motion paths or its sojourn locations, the charging network is assumed to be sparse, i.e., the charging range is much smaller than the distance between charging stations, and at any moment of time, the node can harvest energy from one RF station only. Whenever a node reaches a charging station, it rests for a fixed amount of time and then moves towards a randomly selected charging station with a fixed velocity. A fraction of charging stations is selected as home stations where a node sojourns for a longer time. The sensor duty cycle is set to be equal to the amount of harvested energy, i.e., the sensor operates in energy-neutral mode [16].

The proposed method has been evaluated using a custom simulator written in Python. The sensor node is implemented as an object with the following core methods: charge(), leak(), runTask(), setCapLarge(), setCapSmall(), getVoltage(), monitorColoc(), switchCap(). At every time step, the node moves towards a random waypoint, then charges, leaks, detects and tracks collocation and performs a task with available energy. The simulation parameters such as charging power, leakage and capacitor sizes have been initialised based on prototype measurements described in the following section.

The performance is evaluated through measuring the total amount of usable harvested energy, sensing coverage measured by successful sensor activations and the number of served locations. The performance is compared with a baseline approach, where a fixed capacitor of either a small or large value is used. The simulation parameters are given in Table 1.

### 4.1. Usable Harvested Energy

In mobile scenarios, the node may leave the RF charging station before the capacitor voltage reaches $V_{max}$ required to boot the node, and any residual charge will start to gradually dissipate until the node visits another RF station. In the most extreme scenario, when the node always leaves the RF station before the capacitor is fully charged, all harvested energy will be dissipated between successive partial charges.

We define usable harvested energy as the total amount of harvested energy less the amount of energy spent on charging a capacitor prior to node startups, ignoring the energy harvested while $V_{cap}<V_{max}$. In other words, usable harvested energy is the total amount of harvested energy that is actually available at an application layer, when the device is already active and ready to operate and until the capacitor voltage stays above $V_{cap}>V_{min}$. In the experiment below, we show that usable harvested energy may constitute only a fraction of total harvested energy due to losses, which increase with the capacitor size.

**Figure 4:**
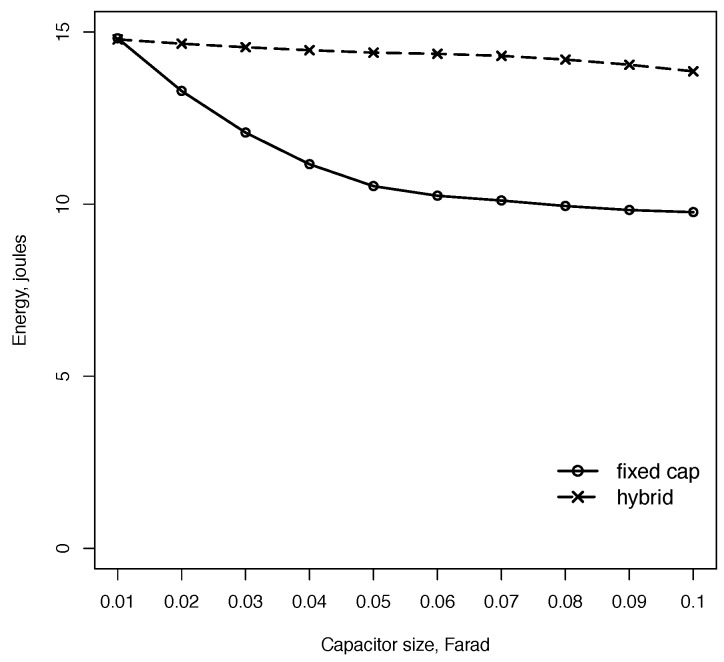
The amount of usable harvested energy in the fixed capacitor configuration decreases with capacitor size due to self-discharge losses between intermittent charges. The hybrid configuration minimizes the losses by using a small capacitor in low energy areas.

The first experiment demonstrates the impact of capacitor size on the total usable harvested energy. The hybrid configuration combined a small 10-mF capacitor with a larger capacitor in the 10–100 mF range. Figure 4 demonstrates that increasing the capacitor size from 10 mF to 100 mF reduces the amount of usable harvested energy by 40%. The hybrid approach in contrast shows consistent performance for various capacitor sizes. The usable harvested energy reduces slightly due to the time needed for an initial capacitor charge when the node switches to a large capacitor near a home station.

### 4.2. Sensor Coverage

**Figure 5:**
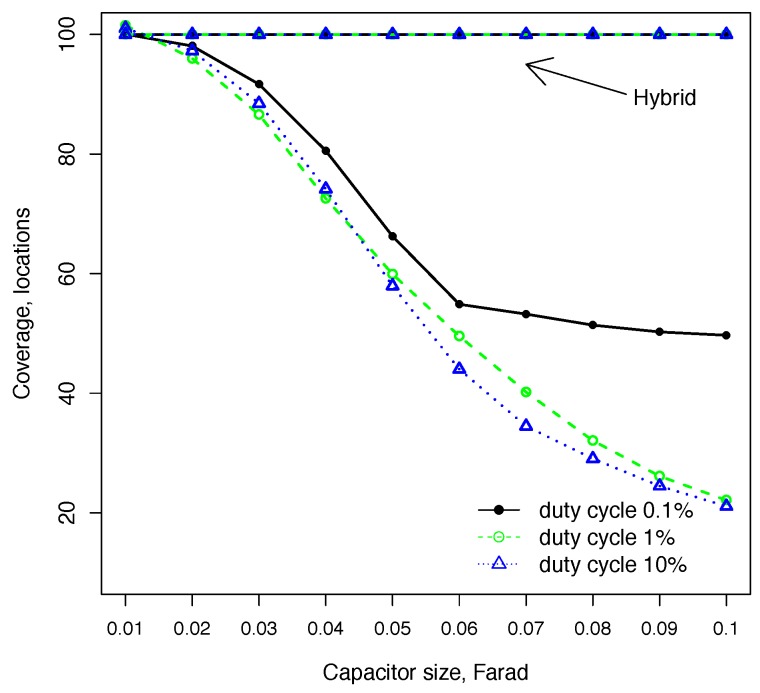
The number of unique locations served by a sensor in the fixed capacitor configuration decreases with capacitor size as the node is less likely to boot on a larger capacitor. The hybrid configuration provides nearly full coverage.

In this experiment, we measure sensor coverage, i.e., a proportion of unique locations where a sensor was able to boot and execute a task, i.e., represents a geographical coverage. The task run by a sensor node can be supported by a small 10-mF capacitor. For a fixed capacitor configuration, the coverage depends on the duty cycle, as shown in Figure 5, with maximum coverage achieved by the lowest duty cycle. Increasing the fixed capacitor size from 10 mF to 100 mF reduces the coverage by 42% at 0.1% duty cycle. Stepping up the duty cycle to 1% further reduces coverage by up to 80% compared to the 10-mF configuration. However, increasing the duty cycle beyond 1% does not have a significant effect on the coverage. The hybrid configuration provides a consistent near 100% coverage at all capacitor sizes. In hybrid configuration, the node boots on a small capacitor, which enables it to boot at all locations. The performance of hybrid configuration does not depend on the larger capacitor size, as it performs the switch only when the available energy is sufficient to charge the larger capacitor.

### 4.3. Sensor Activations

**Figure 6:**
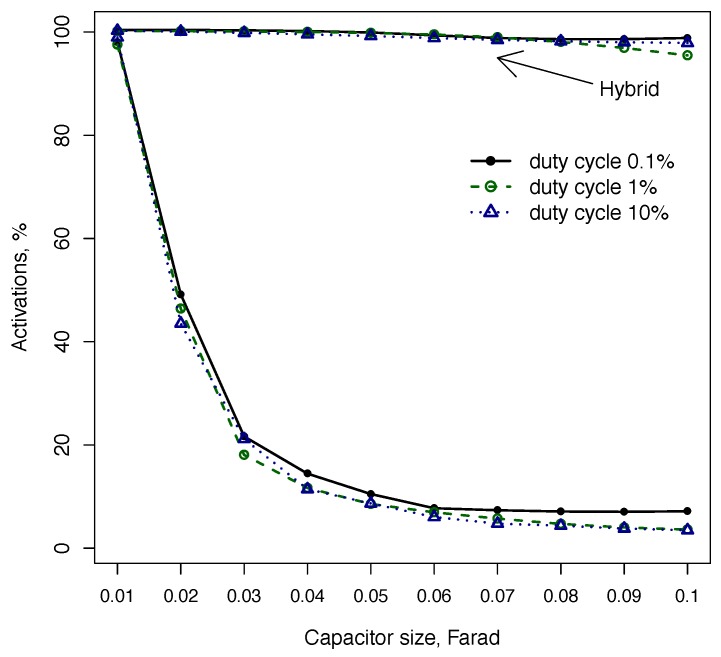
The number of sensor activations near charging stations in the fixed capacitor configuration drops rapidly with capacitor size as the node is less likely to boot on a larger capacitor.

Finally, Figure 6 shows sensor activations defined as the proportion of all (non-unique) locations where a node was able to boot and execute a task. In other words, multiple visits to the same location are counted as separate activations. This represents sensing applications, where the node needs to maximise the total amount of collected data irrespective of locations. It can be seen that a capacitor size has a drastic effect on sensor activations, reducing it by up to 90%. The hybrid configuration is not affected by a capacitor size and consistently achieves 100% activations. The number of sensor activations for hybrid configuration does not depend on the larger capacitor size, as it performs the switch only when the available energy is sufficient to charge the larger capacitor.

### 4.4. Available Energy

Table 2 shows the peak usable energy for hybrid mode within a high energy zone. Combined with the results in Section 4.1–Section 4.3, it demonstrates that the sensor node can achieve efficient energy harvesting with high coverage and activations while supporting a large energy storage, which allows a sensor to execute an energy-intensive task whenever required.

## 5. Prototype

**Figure 7:**
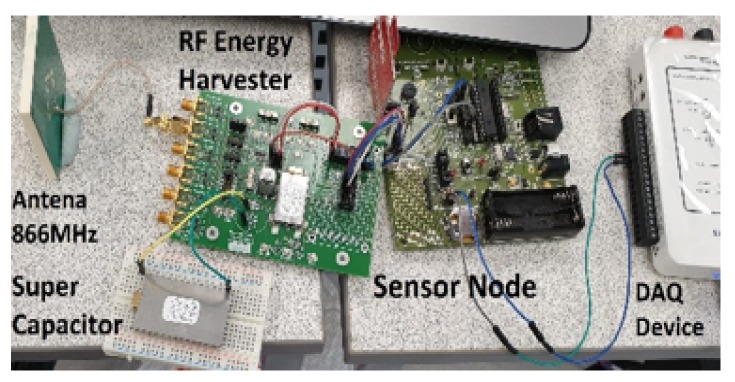
Measurement setup.

To validate the proposed design experimentally, a hybrid RF node design has been implemented based on the PowerCast P21XXCSR-EVB RF Energy Harvesting development kit (Powercast, Pittsburgh, PA, USA), which can harvest energy from six frequency bands and store energy in a built-in 50-mF capacitor or an external capacitor, connected through an extension port; see Figure 7. The 868-MHz ISM band has been selected as it has 865.6–867.6 MHz sub-band, which allows 3-W EIRP transmission in the UK [6]. The 868-MHz RF charging signal has been generated by a Rohde&Schwarz SMC 100 (Rohde&Schwarz, Munich, Germany) A signal generator with an AML 32210 Amp and a 6-dBi transmitting antenna. The board harvests energy from −15 to 15 dBm, provides three output voltage levels of 3.3 V, 4.1 V and 4.2 V and delivers up to 50 mA. With this setup, the board can harvest the energy from a 3-W 866-MHz source at a distance of 11 m. The purpose of this section is to demonstrate that the concept is practical and can be implemented using off-the-shelf hardware with minimum costs rather than reproducing and comparing with large-scale simulation experiments. This is because deployment and measurements within an RF energy harvesting environment over a large geographical area would be extremely challenging in terms of resources and logistics. Developing a case study, where RF energy harvesting platform is used for a real application, and reporting the results are potential future work.

**Figure 8:**
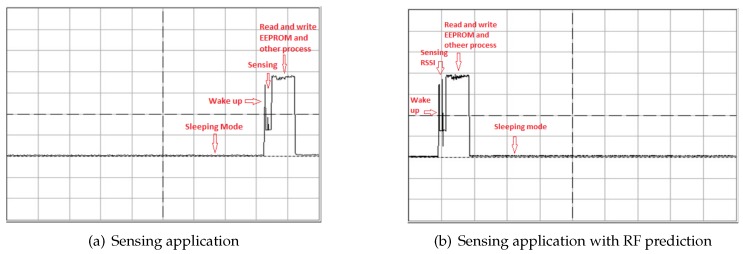
Oscilloscope screenshot for the energy profile of a simple application and the prototype. The oscilloscope was set at 20 mV/div with a timebase of 10 ms/div.

The XLP 16-bit development kit (Microchip Technology, Chandler, AZ, USA) used as the sensor node consists of a PIC24F16K102 microcontroller, the temperature sensor MCP9700 and a MiWi MRF24J40MA short-range low power transceiver. The MCP9700 is a low power thermistor operating at 6 μA. The sensor node is powered through an RF energy harvesting P21XXCSR-EVB board and can change its energy storage through a low power switching circuit that we have designed, based on the P1 bi-stable V23026 relay, operating on 3 V. The algorithm has been implemented in MPLAB X IDE using the C 30 compiler, which produces a 51-kB HEX file with 81% of programming memory allocated for code and 24% of data memory for data. The impact of RF energy prediction on the overall energy consumption has been evaluated by measuring the energy consumption of the basic sensing application and then measuring the energy consumption of the same sensing application, but with the algorithm implemented. The energy consumption was measured by connecting an oscilloscope to the jumper 9 of the XLP development kit and the connecting 22-Ohm shunt resistor in the socket. The energy consumed for the basic sensing task in which the sensor was programmed to sense periodically, save data to flash and sleep was measured as 7.93 $\times \;10^{-5}$ J; whereas when the sensor node executed the RF sensing with energy prediction, the task energy cost increased to 9.2 $\times \;10^{-5}$ J, which represents only a 16% increase due to the need to sense Received Signal Strength (RSSI). Table 3 shows the measured power consumption in different modes. Figure 8 shows the energy profiles of a simple sensing application (a) and a sensing application with an implemented RF prediction algorithm.

### 5.1. Charging Time

**Figure 9:**
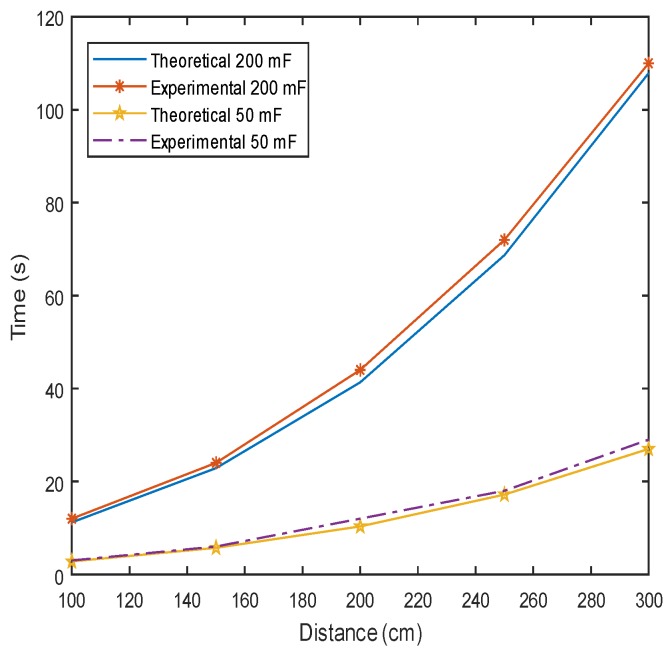
The charging time duration for 50-mF and 200-mF supercapacitors increases exponentially with charging distance. Practical applications that require longer charging range would thus require a very small capacitor.

The goal of the first experiment was to demonstrate the performance of a simple RF energy harvesting application, the impact of capacitor size and the distance on the charging time of a sensor node running a simple application, where a sensor node simply boots and transmits a packet to a base station. The interval between packets is taken as the charging time of the supercapacitor. The upper and lower threshold voltages were set to 1.23 V and 1.02 V, respectively. Figure 9 compares the charging time as a function of distance for 50-mF and 200-mF capacitors. As expected, the amount of time to charge a large capacitor was significantly longer. The amount of time to fully charge a large capacitor at a 3-m distance was 110 s, whereas it took only 29 s for a small capacitor. The figures also compare the experimental results with the theoretical values obtained through the following model [17].

### 5.2. Sensor Node Lifetime

**Figure 10:**
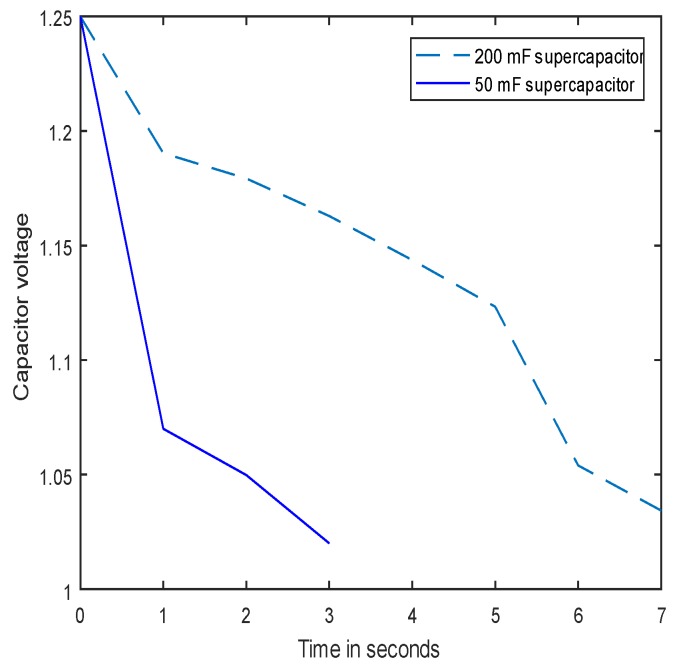
Node lifetime.

The goal of the second experiment was to demonstrate the lifetime of a node running a simple application, which wakes up to sense temperature and then sleeps every 5 s. In this experiment, the harvester board with a 50-mF capacitor is first charged to 1.25 V by a 33-dBm 866-MHz source and then connected to a sensor node with the lower threshold voltage set to 1.02 V. The experiment has been repeated with a 200-mF capacitor. The voltage across the $V_{out}$ and GND terminals of the board have been measured with a multimeter. As can be seen from Figure 10, the maximum sensor lifetime was measured as 3 s and 7 s with 50-mF and 200-mF capacitors, respectively. The experiment was conducted for a single capacitor configuration.

### 5.3. Dynamic Capacitor Switching

In the final experiment, we demonstrate the practical feasibility of the hybrid dual-capacitor configuration for a simple sensing and data collection application, which senses a temperature reading, saves it to a flash storage and transmits wirelessly to the base station. To evaluate the dynamic switching storage, a sensor node was periodically moved from a distance of 3 to 1 m from the RF charging station to emulate low and high energy areas, respectively. The RSSI threshold was set to 2 dBm representing a distance of approximately 2 m, while the sojourn time near the charging station has been set to 40 s. In practice, these parameters are application dependent and can be tuned depending on the mobility of the node and transmit power of charging stations. The sensor node was configured to use the small capacitor at the initial boot. Whenever the node sensed that RSSI as higher than threshold value, it estimated the collocation time and saved it into a flash memory after each visit. After the third visit, the node was able to predict correctly that the collocation time within the high energy area would be sufficient to switch to a large capacitor, and after completing the switch, the node successfully sent the collected data to the base station. When moved out of the high energy area, the node waited until the large capacitor voltage dropped below 1.045 V to switch back to the small capacitor and resume the sensing task. The experiment has been repeated in a single fixed-capacitor configuration of either 50 mF and 200 mF by varying the sojourn time between 40 s and 10 s. With a 40-s sojourn time, both small and large capacitors were able to boot the node; however, only the large one was able to support the transmit operation. When the sojourn time was reduced to 10 s to mimic environments with lower energy, the node has never been able to boot on a large capacitor, but booted successfully on a small one.

## 6. Related Work

Supercapacitors have become an attractive option for energy storage due to high power density, fast charge time and virtually unlimited charge/discharge cycles. The main limitations of supercapacitors are low energy density and relatively high self-discharge current compared to conventional chemical batteries. As the classical conventional supercapacitor circuit model mostly fails to explain its behaviour, [2] proposed an equivalent model, which consists of multiple nonlinear RC branches and explains self-discharge current as electric charge redistribution between slow and fast RC branches. The model shows that the charging has to be long enough to charge the capacitor in the slowest RC branch, as otherwise, even though the supercapacitor appears to be charged, the electric charge from the fastest branch will flow into other branches, which will appear as leakage current from outside. The work in [18] proposed ladder, three-branch, four-branch models. The work in [19,20] proposed an energy iteration model, which explains self-discharge as the only reason for the terminal voltage drops, but ignores the effect of charge-redistribution.

Task scheduling: The resulting models have been used by researchers for optimizing task scheduling. The work in [20] adapted the duty cycle for sensor nodes to evade the working of the supercapacitor in high leakage current regions. The work in [4] investigated the effect of charge redistribution on power management and established that the knowledge of supercapacitor state helps to select a task scheduling policy that makes full use of stored energy. Although the capacitor models can be quite accurate in estimating the behaviour of supercapacitors, they can be quite complex to parameterize due to the need for extensive measurements and model fitting using numerical optimization techniques. The proposed dual capacitor approach does not require the knowledge of the equivalent circuit model and simply relies on the fact that the energy stored in the supercapacitor gradually dissipates. RF energy harvesting: Battery-less systems utilize a variety of energy sources, such as solar, vibration, temperature difference and wireless power transfer. RF energy harvesting is a relatively new technique, which harvests power from RF sources and can power devices in the environments where light, vibration and temperature difference are not available. The RF energy harvesting systems can scavenge ambient RF energy, such as Wi-Fi, or TV, or radio broadcast signal [21], or obtain energy radiated by dedicated RF transmitters. In [22], a battery-less RF sensor has been designed to monitor the quality of packed vegetables by observing their temperature and humidity. Such sensors receive a stable flow of energy from a nearby RFID reader for the entire duration of measurement, which is sufficient to complete the sensing and communication task. In contrast, the proposed approach uses sensors that are autonomous and that opportunistically collect energy from charging stations located in strategic positions, and therefore need to be able to extract and utilize tiny amounts of energy, wherever it is available. The work in [23,24] proposed an RF energy harvesting system that powers low power devices from medium wave radio signals and that can operate forever within relatively large geographical areas, but only in the proximity of a powerful MW radio station. The hybrid approach proposed in this paper however is suitable for any area instrumented by relatively inexpensive and low power custom transmitting stations. The work in [25] analysed the network connectivity problem in a wirelessly-powered battery-less sensor network, which arises from the fact that the nodes are powered intermittently, leading to connectivity problems, and proposed an approach based on dividing time into separate harvesting and communication periods. Similarly to [22,23], the approach relies on the stable and predictable energy flow from the source.

The work in [1] described a federated energy storage concept, where each peripheral, such as radio, sensor or a microcontroller, is assigned a dedicated capacitor for increased reliability so that faulty or misconfigured peripherals do not affect the shared energy storage and thus the operation of the entire system. The platform contains a first-stage capacitor, which powers the microcontroller, an array of peripheral capacitors, and allows for faster charging by setting capacitor sizes for specific peripherals and controlling the charging priority to individual capacitors. In subsequent work, the authors described a reconfigurable federated energy storage, where an engineer can assign charging priorities, capacitor sizes and voltage thresholds in both compile and run-times. The main limitation of federated energy storage is that it requires the first-stage capacitor to be charged to power the microcontroller and the peripheral capacitors. Once the first-stage capacitor is depleted, the microcontroller shuts down, and the energy stored in peripheral capacitors cannot be used and will be slowly dissipated. Similarly to a fixed capacitor design, there is a dilemma in that the first-stage capacitor needs to be small to charge quickly, but large enough to operate an MCU when the node moves to a low energy area, which presents a problem for mobile battery-less applications. In contrast, the proposed hybrid platform is powered by a single capacitor at any moment of time. When the node detects a high energy area, it switches to a bigger capacitor, which enables it to accumulate available energy and use it when the node moves into a low energy area.

## 7. Conclusions and Future Work

Battery-free energy harvesting sensors provide a promising solution for a maintenance-free and perpetual sensor operation. We have proposed a novel approach for hybrid energy storage, which is able to adapt and reconfigure energy storage depending on the environment and resolve an important trade-off between fast start-up time and amount of stored energy. The evaluation based on simulations and the hardware implementation on the PowerCast RF energy harvesting kit have demonstrated that the hybrid platform can operate in environments where fixed capacitor nodes can fail. In particular, the proposed hybrid approach showed up to 40% and 80% performance improvement over a fixed capacitor design in terms of usable harvested energy and the sensor coverage, respectively. We have demonstrated that a traditional single fixed-capacitor design is not suitable for certain RF energy harvesting applications in sparse mobile networks. While we have used a dual-capacitor configuration as an example of a hybrid storage concept, extending the proposed scheme to three or more capacitors may provide applications with more fine-grained control over energy storage. As a typical sensor node has a limited number of output pins for addressing individual capacitors, this would require modifications to the switching circuit and would lead to higher switching cost due to the increased number of relays and other components. Investigating the design trade-offs for various numbers of energy storage devices is a potential future work.

## Figures and Tables

**Table 1:** Simulation parameters.

Parameter	Value
area	3000 m × 3000 m
avg speed	1.38 m/s ± 0.28 m/s
rest time, home station	600 s
rest time, charging station	5 s
leak current	4 μA
sleep current	4 μA
RF power output	3 W
RF min distance	1.5 m
$V_{max}$	1.25 V
$V_{min}$	1.02 V

**Table 2:** Peak usable energy in energy storage near home stations.

**Hybrid Capacitor, F**	0.01	0.02	0.03	0.04	0.05	0.06	0.07	0.08	0.09	0.10
**Energy, mJ**	12.84	25.60	38.44	51.28	64.12	76.95	89.79	102.63	115.47	128.31

**Table 3:** Sensor node power consumption in different modes. The node lifetime increases with capacitor size.

Mode	Current, mA	Voltage, V	Power, mW
sleeping	$1\times 10^{-3}$	3.3	$3.3\times 10^{-3}$
active	3.34	3.3	11.022
transmitting	35.587	3.3	117.44

## References

[1] Hester J., Sorber J. Flicker: Rapid Prototyping for the Batteryless Internet-of-Things. Proceedings of the 15th ACM Conference on Embedded Network Sensor Systems.

[2] Merrett G.V., Weddell A.S. Supercapacitor leakage in energy-harvesting sensor nodes: Fact or fiction?. Proceedings of the 2012 Ninth International Conference on Networked Sensing (INSS).

[3] Zhang Y., Yang H. (2011). Modeling and characterization of supercapacitors for wireless sensor network applications. J. Power Sources.

[4] Yang H., Zhang Y. (2013). Analysis of Supercapacitor Energy Loss for Power Management in Environmentally Powered Wireless Sensor Nodes. IEEE Trans. Power Electron..

[5] Gummeson J., Clark S.S., Fu K., Ganesan D. (2010). On the Limits of Effective Hybrid Micro energy Harvesting on Mobile CRFID Sensors. Proceedings of the 8th International Conference on Mobile Systems, Applications, and Services.

[6] Powercast (2018). P21XX Powerharvester Chipset Reference Design Evaluation Board.

[7] Merz C., Kupris G., Niedernhuber M. Design and optimization of a radio frequency energy harvesting system for energizing low power devices. Proceedings of the 2014 International Conference on Applied Electronics.

[8] Calabrese F., Diao M., Lorenzo G.D., Ferreira J., Ratti C. (2013). Understanding individual mobility patterns from urban sensing data: A mobile phone trace example. Transp. Res. Part C Emerg. Technol..

[9] Ellwood S., Newman C., Montgomery R., Nicosia V., Buesching C., Markham A., Mascolo C., Trigoni N., Pasztor B., Dyo V. (2017). An active radio frequency identification system capable of identifying co locations and social structure: Validation with a wild free ranging animal. Methods Ecol. Evol..

[10] Musolesi M., Mascolo C. (2008). CAR: Context-Aware Adaptive Routing for Delay-Tolerant Mobile Networks. IEEE Trans. Mob. Comput..

[11] Lindgren A., Mascolo C., Lonergan M., McConnell B. Seal-2-Seal: A delay-tolerant protocol for contact logging in wildlife monitoring sensor networks. Proceedings of the 2008 5th IEEE International Conference on Mobile Ad Hoc and Sensor Systems.

[12] Noulas A., Scellato S., Lathia N., Mascolo C. Mining User Mobility Features for Next Place Prediction in Location-Based Services. Proceedings of the 2012 IEEE 12th International Conference on Data Mining.

[13] Hristova D., Noulas A., Brown C., Musolesi M., Mascolo C. (2016). A multilayer approach to multiplexity and link prediction in online geo-social networks. EPJ Data Sci..

[14] Kalman R.E. (1960). A New Approach to Linear Filtering and Prediction Problems. Trans. ASME J. Basic Eng..

[15] Grewal M.S., Andrews A.P. (2008). Kalman Filtering: Theory and Practice Using MATLAB.

[16] Savanth A., Bellanger M., Weddell A., Myers J., Kauer M. (2018). Energy neutral sensor system with micro-scale photovoltaic and thermoelectric energy harvesting. J. Phys. Conf. Ser..

[17] Mishra D., De S., Chowdhury K.R. (2015). Charging Time Characterization for Wireless RF Energy Transfer. IEEE Trans. Circuits Syst. II Express Briefs.

[18] Faranda R. (2010). A new parameters identification procedure for simplified double layer capacitor two-branch model. Electr. Power Syst. Res..

[19] Zhu T., Zhong Z., Gu Y., He T., Zhang Z.L. (2009). Leakage-aware Energy Synchronization for Wireless Sensor Networks. Proceedings of the 7th International Conference on Mobile Systems, Applications, and Services.

[20] Zhu T., Zhong Z., He T., Zhang Z.L. (2013). Energy-synchronized computing for sustainable sensor networks. Ad Hoc Netw..

[21] Alneyadi F., Alkaabi M., Alketbi S., Hajraf S., Ramzan R. 2.4 GHz WLAN RF energy harvester for passive indoor sensor nodes. Proceedings of the 2014 IEEE International Conference on Semiconductor Electronics (ICSE2014).

[22] Le G.T., Tran T.V., Lee H.S., Chung W.Y. (2016). Long-range battery-less RF sensor for monitoring the freshness of packaged vegetables. Sens. Actuators A Phys..

[23] Ajmal T., Dyo V., Allen B., Ivanov I. (2014). Design and optimisation of compact RF energy harvesting device for smart applications. Electron. Lett..

[24] Dyo V., Ajmal T., Allen B., Jazani D., Ivanov I. (2013). Design of a ferrite rod antenna for harvesting energy from medium wave broadcast signals. J. Eng..

[25] Mekikis P.V., Antonopoulos A., Kartsakli E., Alonso L., Verikoukis C. Connectivity Analysis in Wireless-Powered Sensor Networks with Battery-Less Devices. Proceedings of the 2016 IEEE Global Communications Conference (GLOBECOM).

